# Understanding empowerment for a healthy dietary intake during pregnancy

**DOI:** 10.1080/17482631.2020.1857550

**Published:** 2020-12-14

**Authors:** Sabina Super, Annemarie Wagemakers

**Affiliations:** Health and Society, Social Sciences Group, Wageningen University, Wageningen, The Netherlands

**Keywords:** Pregnancy, empowerment, dietary intake, antenatal care, midwife, nutritionist, dietitian

## Abstract

**Purpose**: In order for health professionals to encourage pregnant women with a low socioeconomic status (SES) to move towards empowerment for a healthy dietary intake, crucial steps are to understand the perspectives of pregnant women of food and eating and to identify opportunities for empowerment. This study aimed to examine the perspectives of pregnant women on food and eating and to identify the opportunities for empowerment towards a healthy dietary intake.

**Methods**: This was a qualitative participatory study. Thirteen semi-structured interviews were conducted with low SES pregnant women in the Netherlands and analysed using an inductive approach.

**Results**: Five perspectives on food and eating emerged: 1) health-promoting foods and products, 2) challenges in healthy eating, 3) risky products, 4) strategies for healthy eating, and 5) motivational aspects. Opportunities for empowerment were: searching balanced and personalized information, developing strategies to implement healthy diets, navigating the social environment, and dealing with different motivations.

**Conclusion**: Pregnant women hold diverse perspectives regarding food and eating, signalling the need to adjust dietary support from health-care professionals. Professionals in antenatal care could optimize their interactions by engaging in pregnant women’s empowerment processes to make healthy modifications to their dietary intake.

## Introduction

A healthy dietary intake during pregnancy is important for maternal and child health (World Health Organization, 2018) which has been defined by the World Health Organization (2018) as one that “contains adequate energy, protein, vitamins and minerals, obtained through the consumption of a variety of foods, including green and orange vegetables, meat, fish, beans, nuts, pasteurized dairy products and fruit (p. 4)”. A poor dietary intake is associated with adverse health outcomes for the mother (e.g., increased risk of pre-eclampsia and gestational diabetes, and excessive gestational weight gain), adverse birth outcomes (e.g., premature birth and low birth weight), and adverse health outcomes in childhood and adult life (e.g., increased risk of developing chronic diseases such as diabetes or coronary heart disease) (James-McAlpine et al., 2020; Ramakrishnan et al., 2012; Stang & Huffman, 2016).

Pregnant women are often motivated to consume a healthy dietary intake during pregnancy (Lindqvist et al., 2017) and are generally satisfied with their dietary patterns, although they often do not meet nutritional guidelines (Malek et al., 2016). It has been argued that pregnancy is a teachable moment, where women are open to lifestyle changes as they gain awareness of the importance of health behaviours for foetal health (Lindqvist et al., 2017; Szwajcer et al., 2012). However, they may encounter challenges (e.g., nausea) that make it difficult to implement and sustain dietary changes throughout pregnancy (Blumfield et al., 2012; Malek et al., 2016). Pregnant women with a low Socio-Economic Status (SES) face additional challenges that complicate having a healthy dietary intake, such as a lack of money, knowledge and skills to make dietary changes (Baron et al., 2015; Fowles et al., 2011; Renzaho & Oldroyd, 2014).

Midwives play an important role in supporting pregnant women to choose a healthy dietary intake, but their advice is often presented in very general terms and primarily focused on food safety issues only (Arrish et al., 2014; Bryant et al., 2019; Garnweidner et al., 2013). Midwives often experience a lack sufficient time and resources to provide adequate nutritional support (McCann et al., 2018), which is problematic when working with low SES pregnant women who often face significant challenges to implementing a healthy diet. Nutritional support from midwives could be improved to empower low SES pregnant women to have a healthier dietary intake, while at the same time taking into account the limitations in time and resources that midwives face in providing this support.

Definitions of empowerment are not equivocal, but in this paper we follow the definition provided by Koelen and Lindström (2005): “*Empowerment is a process by which people gain mastery (control) over their lives, by which they learn to see a closer correspondence between their goals and a sense of how to achieve these goals, and by which people learn to see a relationship between their efforts and the outcomes thereof* (p. 12)”. In order for health professionals, such as midwives, to encourage pregnant women to move towards empowerment for a healthy dietary intake, it is crucial to gain in-depth understanding of low SES pregnant women’s perspectives of food and eating during pregnancy. At the core of empowerment is the idea that health professionals use a bottom-up strategy to help support people on issues and problems that are defined by the people themselves, and so these perspectives form the starting point of health professionals’ efforts in enabling people towards empowerment (Tengland, 2012). A study by Copelton (2007) showed that highly educated and affluent women linked healthful eating in pregnancy to ideals of being a good mother. Pregnant women also used several justification strategies to account for deviant nutritional behaviour. For example, women tended to deny the potentially harmful consequences of unhealthy foods or claimed that nutritional guidelines for pregnant women posed unrealistic standards (Copelton, 2007). In a study among Australian women with post-secondary education (Lucas et al., 2016), it was found that women had a strong desire to do what was good for their baby and showed high-risk aversion in relation to eating fish and seafood during pregnancy. However, this contrasts with guidelines that encourage low mercury and sufficiently cooked fish and seafood consumption for foetal growth and development (European Food Safety Authority, 2014). Finally, it has also been demonstrated that pregnant women experience confusion regarding food and eating when navigating conflicting and sometimes incorrect sources of nutrition information, making it difficult to make healthy choices (Bianchi et al., 2016; Bookari et al., 2017; Garnweidner et al., 2013). While these studies suggest that perspectives on and beliefs about food and eating are important for the dietary intake of pregnant women, qualitative studies investigating the meaning of healthy eating for low SES pregnant women are currently lacking. As such, the first aim of this study is to examine the perspectives of low SES pregnant women on food and eating during pregnancy.

In the process of empowerment, it is not only important to involve people in defining the problem or issue, but also to actively involve them in finding solutions to these problems and the actions that are needed to tackle them (Tengland, 2012). Empowerment is not something that can be imposed on people, but something that is sought and gained by people themselves (Laverack, 2006). Empowerment is about gaining mastery and learning how to achieve goals that are meaningful to the individual or to the community (Koelen & Lindström, 2005). In this process, it is the role of the health professional to support people by enabling reflection on barriers and opportunities for change and strengthening one’s capacity to make those changes (Koelen & Lindström, 2005). It has been demonstrated that high SES pregnant women face both individual and environmental barriers towards healthy eating during pregnancy (Bookari et al., 2017), requiring the interactive engagement of health care providers with pregnant women to not only provide factual and practical information, but also to discuss, reflect and comment on the performance of women in relation to their dietary intake. In this learning process, the focus should be on enablers for a healthy dietary intake and the strengths and competencies already possessed by pregnant women to improve or sustain a healthy diet. As research in the area of empowerment towards a healthy dietary intake in pregnancy is sparse (Bookari et al., 2017; Brandstetter et al., 2015), the second aim of this study is to identify the opportunities for empowerment towards a healthy dietary intake amongst low SES pregnant women.

In sum, this study aims to answer the following research questions using multiple qualitative methods of data collection and analysis (i.e., card sorting task, content analysis, counts and inductive thematic analysis):

1. What are the perspectives of low SES pregnant women on food and eating during pregnancy?
2. What are the opportunities for empowerment towards a healthy dietary intake amongst low SES pregnant women?

## Participants, ethics and methods

### Study design

This study is part of a multi-stage research project “Power 4 a Healthy Pregnancy” aiming to develop and implement an integral strategy in antenatal care to empower low SES pregnant women to have a healthier dietary intake (Beulen et al., 2020). Action research is at the core of the research project to ensure that the strategy is developed not only for but in strong collaboration with the stakeholders (i.e. low SES pregnant women, midwives, dietitians, gynaecologists and other health professionals). In this way, the strategy closely matches the needs, interests, and capacities of the different stakeholders involved (Goodman & Sanders Thompson, 2017; Israel et al., 1998). The current study encompasses the first phase of the research project, which was to explore the perspectives of low SES pregnant women on food and eating during pregnancy and to understand the opportunities for empowerment as experienced by these women.

The research activities were developed in close collaboration with pregnant women and young mothers in co-design meetings. This process was followed to enhance the relevance and usefulness of the research activities for the target group and ultimately improve the quality and validity of the data collection and analysis (Israel et al., 1998). Prior to the two co-design meetings, the two research questions (as formulated above) were defined by the research team. In addition, the research team had decided that in-depth interviews would be used as a data collection strategy. Two co-design meetings were organized to inventory the topics that needed to be addressed in the in-depth interviews and to discuss the most suitable approach for interviewing pregnant women with a low SES. One of the co-design meetings was organized with the Parents Advisory Board [In Dutch: Ouderadviesraad], a board of mothers that advises on and advocates for the needs of expecting and young parents in antenatal care (n = 3). The second co-design meeting was organized in collaboration with a local midwifery practice, which recruited low SES pregnant women (n = 3) to participate. During the co-design meetings, the participants were asked to list important themes that should be addressed in the interviews and formulate interview questions that would be applicable and understandable for low SES pregnant women. The co-design meetings were facilitated by the lead researcher. Insights that formed the basis for the in-depth interviews to be conducted with low SES pregnant women were:

- Perspectives on food and eating may be diverse. Making a mind map allows for creative thinking as “anything goes”.
- Food and eating during pregnancy are potentially sensitive topics. Interviewers should be careful not to judge or comment on the dietary intake of pregnant women. Avoid testing nutrition knowledge and behaviour or talking about the urgency/importance of healthy eating during the interviews.
- An open and supportive environment during the interview is important to make sure that the women feel safe to present their view on food and eating during pregnancy.

### Participants and procedure

In-depth interviews were conducted with 13 low SES pregnant women, defined as women following or having completed lower secondary education and/or women with a low household income which was subjectively assessed by the midwives. No inclusion criteria were formulated for parity and marital status. Pregnant women were recruited via midwives collaborating within the research project and contacted by the lead researcher to schedule an interview. The participants signed an informed consent form stipulating their right to stop the interview at any point, guaranteeing the confidential use of the data in the study, and describing the protocol for data storage and sharing. The interviews took place at a preferred location by the participating women and lasted on average 46 minutes (between 24 and 56 minutes). In two cases, the partner of the pregnant woman was present during the interview. Recruitment ceased when data saturation, as determined by researchers, was reached. Women received a gift voucher of 20 euros for participation. This research was conducted by two female researchers (SS and AW) trained in qualitative research methods and experienced in conducting research amongst low SES groups. One researcher (SS) has a background in health promotion, with a specific focus on promoting health and wellbeing in vulnerable groups, and one researcher (AW) has wide experience with complex public health-promotion projects. Her research focuses on the combined influence of lifestyle and the social and physical environments of health and well-being in real-life settings. This study was approved by the Social Science Ethics Committee (SEC) of the Wageningen School of Social Sciences.

The in-depth semi-structured interviews were conducted by one researcher (SS) and consisted of four parts (see Table I). The interviews started with a cluster of background questions, including family situation, experiences with previous pregnancies (when applicable), context of current pregnancy (positive and negative moments), experiences of social support, contact with gynaecologist or dietitian (when applicable), and changes that have been made in current or previous pregnancies with regards to food and eating. The second part of the interview was directed at understanding the perspectives of low SES pregnant women of food and eating during pregnancy. Women were asked to brainstorm about anything that came to mind when hearing “food and eating during pregnancy”. Either the interviewer or the participant noted all the ideas on a large sheet of paper. The interviewer asked follow-up questions to better understand the ideas put forward by the women, for example by asking for illustrations of a mentioned phenomenon or by asking for specific situations in which the phenomenon proved important in the experiences of the participant.

**Table I.** Overview of the interview guide

Block number	Purpose/content	Example questions
1	Obtaining background information	What is your family situation? What changes have you made to your diet since you became pregnant? What has been the most difficult moment during your pregnancy so far?
2	Understanding the perspectives of low SES pregnant women of food and eating during pregnancy	I would like you to brainstorm about the topic “food and eating during pregnancy”. What topics come to mind? Can you give an example of this [answer given by pregnant women during brainstorm]?
3	Identifying opportunities for empowerment towards a healthy dietary intake amongst pregnant women	For which of these product groups do you think you are following the dietary guidelines? For which of these product groups do you think you might eat more healthy? What would help you to eat more healthy when it comes to this specific product group?
4	Reflecting on the interview and identifying lessons learned	Looking back at this interview, have you learned anything new? Looking back at this interview, what would you recommend other pregnant women to eat more healthy during pregnancy?

The third part of the interview aimed to identify opportunities for empowerment towards a healthy dietary intake amongst pregnant women. The participants received nine cards containing pictures of important product groups (i.e., vegetables, fruits, whole wheat products, nuts, dairy, raw meat, fish, sweat/savoury snacks, and alcohol). For each of the product groups, the participants were asked to identify 1) whether they felt they followed dietary guidelines for that product group; 2) whether they would like to improve dietary intake for that product group; 3) what would be challenging to improve dietary intake for that product group; and 4) what would help them to improve dietary intake for that product group. The cards contained pictures of the food groups and the requirements for a healthy diet according to the Health Council of the Netherlands (2015) were printed on the backside. The requirements were not part of the interview to avoid participating women feeling as though they were being judged. Rather, the requirements were only discussed if the participant brought up questions on how much should be eaten from a specific product group. The product groups were chosen based on research conducted in the Netherlands identifying significant differences in dietary intake between low and high SES pregnant women (Beulen et al., 2019). Low SES pregnant women scored significantly worse on the aforementioned product groups than high SES women, signalling the relevance of these product groups for differences in diet quality between high and low SES groups. In the fourth part of the interview, the interviewer reflected with the participants on the interview to identify lessons learned.

### Data analysis

Each participant was assigned a participant number to guarantee anonymity (see Table II). The audio recordings were transcribed verbatim and analysed using Atlas.ti software for qualitative data analysis. The data analysis was performed in two consecutive phases by two researchers (SS and AW) to increase the validity of the results.

**Table II.** Background characteristics of participating pregnant women

Participant number	Age	Number of weeks pregnant	Parity	Family situation
P1	36	13	1	Living with partner and child
P2	27	37	0	Living with partner
P3	25	12	0	Living with partner, partner present during interview
P4	32	Gave birth 2 weeks before the interview	1	Living with partner and child
P5	27	25	0	Living with parents, moving in with boyfriend in near future
P6	22	20	1	Single, living with child
P7	31	13	0	Living with partner
P8	31	38	1	Living with partner and child
P9	35	27	0	Living with partner
P10	22	26	0	Living with partner
P11	30	30	1	Single, child lives in foster care
P12	27	29	2	Single, living with children
P13	39	12	0	Living with partner, partner present during interview

For the first research question, a qualitative content analysis was conducted on the mind maps that were drawn during the interviews. In the first step, the ideas that were raised by the participants were coded as manifest content (i.e., the actual words used by participants) in an inductive manner, thereby staying close to the participants’ lived experiences (Graneheim et al., 2017). This step resulted in 87 codes relating to the participants’ perspectives of food and eating during pregnancy. In the second step, overlapping codes were grouped together to form a single manifest code. In the third step, moving towards a more distant and interpretative approach of qualitative content analysis, the manifest codes were reviewed to identify common themes. These themes addressed the latent content (i.e., the symbolic meaning) of the mind maps and are presented in the results section. Steps 1 to 3 are graphically displayed in the supplementary materials.

For the second research question, an inductive thematic analysis was conducted following the guidelines of Braun and Clarke (2006) and using the concepts of empowerment as provided in the definition by Koelen and Lindström (2005). The analysis focused on the challenges that the participants were confronted with when trying to eat healthy foods and the strategies that the participants shared for consuming and promoting healthy eating during pregnancy. Initial codes were generated that reflected opportunities for promoting healthy eating during pregnancy and resources that could be used by the pregnant women to eat healthier. Differences in codes between primi- and multiparous women were investigated, as well as between single and married/cohabitating women. These codes were then reviewed to identify recurring themes which are presented in the results section. To identify opportunities for empowerment, specific attention was paid in the analysis to the 4^th^ part of the interview in which the interviewer reflected with the participants on the interviews. Moreover, in a joint reflection, the two researchers reviewed the role of the interview in the process of empowerment, as it seemed that an empowerment process took place during the interviews. In this process, the researchers critically reflected on the field notes of the interviewer and the reflections between interviewer and participant.

## Results

The results section is divided into three sections. First, the results of the mind maps are presented to identify the perspectives of low SES pregnant women of food and eating during pregnancy. Next, the results are presented regarding the opportunities for empowerment towards a healthy dietary intake for low SES pregnant women. Finally, reflections on the interviews that are relevant for understanding opportunities for empowerment are offered by the researchers.

### Perspectives on food and eating during pregnancy

Five perspectives were identified during the brainstorm with pregnant women regarding food and eating during pregnancy. The perspectives are discussed in order of the number of appearances during the brainstorm. The perspective that was mentioned most frequently was addressing *health-promoting foods and products*. Women mentioned foods and products that they thought were good for the health of the baby, including orange juice, nuts, whole grain bread, fruits and vegetables, and supplements (e.g., fish oil). The second perspective related to *challenges in relation to eating during pregnancy*. The challenges could relate to a) physical symptoms (e.g., iron deficiency, nausea, hormones), b) mental aspects (e.g., cravings, being moody), and c) other barriers such as difficult social situations (e.g., going out for dinner), contradicting information on the internet and being a vegetarian. The third perspective that appeared during the brainstorm was addressing *risky foods*. Women mentioned an abundance of products they thought they should avoid that could potentially harm the health of the baby, such as alcohol, raw meat, fish, cheese, and energy drinks. This perspective also included mention of specific risks such as “catching bacteria”. The fourth perspective related to *strategies for healthy eating*. These strategies included meal-time strategies to ensure eating healthfully, for example consuming a varied and balanced diet or reducing portion size and increasing frequency of mealtimes. In addition, broader strategies to make it easier for women to eat healthfully were mentioned, such as planning mealtimes with family, asking support of partner or searching the internet for information about healthy eating. The fifth perspective that emerged during the brainstorm related to *motivational aspects* of food and eating during pregnancy. Women indicated that they were highly motivated for healthy eating because of the responsibility they felt for the health of the baby. However, women also mentioned, the difficulties of maintaining motivation as in the case when certain preferred foods are off-limit.

### Opportunities for empowerment

1. Searching for balanced and personalized information

Most participants found it important to receive information on food products that should be avoided and products that promote the health of the baby. However, information received from the midwife about food and eating was often limited to a leaflet that was provided as part of the information package during intake or to having their questions answered. Most women resorted to the internet as a source of information, but indicated that there was conflicting information on the web which made it complicated to judge what was trustworthy. One pregnant woman said:

On the internet is so much information, around all kinds of pregnancy topics. Yes, that can be very conflicting, in any case very confusing on how and what. Some things are very clear, everybody knows that, about alcohol or raw meat. But no, it is quite difficult to say something about mozzarella or brie or liverwurst or tuna […]. You can find conflicting information about that. (P8)

Another source of information on food and eating during pregnancy was the social environment of the pregnant women. Mothers, husbands, and friends provided information on what was healthy or not healthy for the mother and the child. For some women, this was helpful, especially if other sources of information were not present. For others, however this information was also a source of confusion. This was demonstrated by a pregnant woman with Uzbek roots:

Oh my mother is so funny when I tell her that I can’t eat raw fish or meat and I can’t eat that. She is so surprised, she says: “during my pregnancy I could eat everything, as much as I could, nobody monitored me”. But then I say, mom, that is not the case here in the Netherlands, people closely monitor what you eat. And I also feel that I should do that [eat healthy]. For example, she [my mother] says that in Korea or in Japan people eat raw fish. Raw fish with herbs that are really spicy. She says: “they eat that every day”. I said: ”Mom you do not exactly know that. I do not want to take any risks, that is not what I want”. (P2)

Most participating women preferred to have an up-to-date digital list of products stating whether a product should be avoided or not. As a newly launched application by the Dutch Nutrition Centre (2019) was specifically targeting pregnant women, most women referred to this source as trustworthy. However, even though this list of products was useful to judge whether there were any risks associated with a specific product, it did not include any information on health-promoting products or what should be eaten to stimulate the healthy development of a child. When discussing the different product groups (i.e., the nine cards discussed in the interview methods), the participating women indicated they received new information from the interviewer about health-promoting products and on the recommended daily intake per product group. Some women even indicated during the interview that they would like to try to make a dietary change based on this information. For example in reflecting on the interview and the lessons learned one of the participants said:

No, but I have not thought that much about wholegrain products before, so that gives me the idea that perhaps I should eat a little more wholegrain products. Or maybe try something new during pregnancy. (P2)

Importantly, several women indicated that they would not only like to receive information on what should be consumed or avoided, but also information on why this is advised. During the interviews, it became clear that the danger of only providing recommendations without explanation is that the message comes across as paternalistic or forced. Several women indicated that they wanted to be able to judge for themselves whether to follow specific advice and to make their own decisions based on sound and valid information. For example, during one of the interviews, a participant discovered that it was recommended that she increased her dairy consumption. When asked what could help her in increasing her dairy consumption she responded:

I think, indicate how much dairy you need and why it is good for you. Of course I can also look it up myself, but I haven’t done that. If I know why it is good for me, than I have a stronger motivation to do so. Then I just know, like with fibres, then I know that I will have good results with that, that it just works. Like with nuts, you know why it is healthy, and with fruits. But for dairy I did not really know that. (P10)

Finally, the pregnant women made a strong case for personalizing the information they received from health professionals by taking into account their food preferences, food challenges (e.g., nausea or cravings), cultural aspects, and family situations. Some women indicated that they had a strong dislike for certain products (e.g., dairy, vegetables, fish) which were recommended for the health of the baby and they would like to receive information on alternative foods to obtain the required vitamins, minerals, and fibres. One woman mentioned that a dietitian could support her in making a personalized plan that would translate recommendations into products that she could actually consume:

During the intake she [the midwife] said that her husband was also familiar with a gluten free diet. Then the midwife said: “we as midwives cannot really help you, so I would advise you to go to a dietitian again. And now that you are pregnant you should carefully look at your situation again”. I have not done that yet, but I think it is a good one indeed, because it is so specific to me. (P7)

(2) *Developing strategies for implementing healthy diets*

The pregnant women in this study all expressed a strong awareness of the importance of healthy eating for the health of the baby and felt responsible for eating healthy. Yet, the average grade they gave their own diet was a seven out of ten, indicating there was still room for improvement in translating healthy intentions to healthy dietary behaviour. Hence, an important area for empowerment is supporting pregnant women in performing and sustaining those behaviours that they would like to implement. Several women struggled with physical complaints due to pregnancy and were searching for ways to maintain a healthy diet despite these difficulties. Nausea was the most frequently mentioned barrier to healthy eating and several women expressed concerns about their consumption of vitamins and minerals:

Yes, but still, it is not healthy. I feel that my body is not absorbing it all. And I find that difficult to let go of, you know? Then I am thinking … ah no I threw it all up, again nothing remains in my stomach. While you are having such good intentions. (P3)

Similarly, some participants struggled with eating enough vegetables and fruits because they developed an aversion to these foods while being pregnant. While discussing these problems during the interviews, new ideas on how these aversions could be overcome were developed in the interaction between the interviewer and the participant. For example, one woman raised the idea to use vegetables in smoothies, because she liked to consume dairy:

Yes I thought that was very annoying [dislike for vegetables], because especially vegetables are so important. So then I need to start to think about alternatives. By eating more fruits during the day, make smoothies, that you could just drink them. Spread across the day. (P7)

Practical suggestions for how to eat healthy were appreciated by the pregnant women. For example, how to make sure that you eat healthy during work? Or how to use health-promoting products, often with the connotation of “awful tasting”, if one’s partner or other family members are picky eaters? Some women suggested to offer recipes to pregnant women to make cooking easier or to exchange tips on healthy eating at work. For instance, a maternity nurse who was experiencing extreme nausea, spread crackers around the house where she was working. In these ways she could easily grab a cracker during work and was constantly reminded of taking something to eat during her busy day:

Or I spread crackers around the house and take one of those crackers every now and then. One of those dry crackers in between activities. Yes, in the baby room, in the kitchen, any place that I am walking by … I try to keep on track by doing that. (P3)

(3) *Navigating the social environment*

The social environment of the pregnant women also played an important role in food and eating during pregnancy. As already indicated under the first theme, the social environment was a source of, sometimes conflicting, information. More importantly, the support of one’s partner and family was important in eating healthfully throughout pregnancy. In an interview in which the partner of the pregnant woman was present, the role of the partner in the dietary intake of his pregnant wife became very explicit. In this specific case, the partner repeatedly encouraged the woman to make healthy dietary changes, supported her in finding practical solutions for pregnancy-related issues (e.g., nausea), and stressed the importance of a healthy diet for the baby. Below is an excerpt from the interview in which the interviewer (I), the pregnant woman (W), and her partner (P) discuss their views on eating during pregnancy:

*I:* And you [pregnant women] just said that you tried to eat normal just like before you were pregnant. Do you think of this [eating pattern] as being something positive or negative?

*W:* I think it is kind of positive, because I was really afraid of those food cravings, eating candies etc. You know, you want your baby to be as healthy as possible […] Of course that is not only dependent on what you eat, but you do see babies that are born the size of toddlers.

*P:* Yes, but if you can reduce the risk …

*W:* Yes, I do feel that it is a positive thing …

*P:* Even though the chances are bigger, you did still live healthy in those nine months, and then you cannot blame yourself. And the baby, most likely, will be healthy as well. I am more strict on that then her [pregnant women] and it also helps that I am cooking. Yes, I can be very strict. When we were talking about having children I always said to her that we would first lose weight.

*I:* And what gives you the motivation to be so strict on that?

*P:* I have always had a few extra kilo’s. If I don’t pay attention, I will become like this [big gesture]. […] And yes, I have always put on the brakes. But of course I also like to drink beer or wine, delicious foods. But that is part of my life, put on the brakes. So I think, concerning that little baby, he is not choosing to be stuffed.

*I:* And do you have an idea why you are able to put on the brakes? Because I assume that it is not an easy thing to do?

*P:* No it is not easy, that is right. For me it is just personal, I absolutely do not want that. […] I know that I will never be super thin, but as I am now is just fine and I will stick to that. When I met you [pregnant women], you lost about 10 kilo’s or so. Because of my strict diet.

*W:* Yes, I am much less … so I do need that. (P3)

Although the above excerpt shows that the partner can be a strong enabler and supporter of healthy eating, it also appeared at times that the partner can act in a disempowering way. Below is a second excerpt from the interview in which the partner seems to judge the pregnant woman’s distaste for vegetables and concludes with a discouraging statement that change is not going to happen on this issue:

*I:* And if you would like to eat a bit more healthy, what could be the first step?

*W:* I hope that I can eat a bit more vegetables.

*P:* I would know what to do. You [pregnant woman] are a picky eater when it comes to vegetables. I really like to stir fry vegetables, really fresh vegetables etc. I can also, with this kind of [hot] weather, enjoy a meal salad. She does not like that. So if you ask me what could be done better, then I would say …

*W:* Yes, but you cannot change your appetite.

*P:* She did not like vegetables either before pregnancy, and now she is pregnant with all that throwing up. I think when I will do that [cook more vegetables] she will throw up even more. But that would be the thing, in my view, more fresh vegetables. But that is not going to happen anyway. (P3)

Frequently mentioned challenges for healthy eating were social events and situations. Enjoying dinner with family or friends can be difficult when one is confronted with delicious foods that should be avoided. One woman indicated that it was common for her to go out for dinner with family and order a beer or a glass of wine, which made it challenging to refrain from drinking alcohol. In a similar case, a participant talked about a restaurant experience in which the chef refused to serve steak well done:

The only thing that I wanted to add is that going out for dinner when you are pregnant is no fun. I cannot eat half of what is on the menu, maybe three quarters of it. And you still pay a lot. And you always have a discussion with the waiter about that you want it well-done, and they are not offering it well-done. So that means that only the vegetarian meals are an option, but that is not the reason for me to go out for dinner. (P8)

Several women raised concerns about the social norm of healthy eating during pregnancy and how it made them feel monitored and scrutinized by others. An example is given by a woman that joined a local festival and walked around with an alcohol-free beer:

Yes, last Saturday we had a party. I was walking around with a beer. Well, I got approached by at least three people … but I told them … it is just 0% alcohol. But people were looking at me. You see them looking at you like … what is this for kind of person … ? (P8)

In these challenging social situations, the partner can again make an important effort to support the pregnant woman, as demonstrated by one woman who said:

He [husband] also sees that it is difficult to eat gluten free and to cook healthy and nice meals. So he really thinks along, when we go out for dinner he does some research or he calls the restaurant upfront … like: “hey, are you familiar with a gluten free diet. And bingo, she is pregnant as well, is that possible?” He does take responsibility. I think that is very sweet. Then I don’t have to be the complaining woman, but he already did that for me. (P7)

1. Dealing with different motivations

As described in theme 2, the pregnant women gave their own diet a rating of seven (out of ten) and were able to identify areas in which they could make improvements. At the same time, the participating women frequently indicated that they were satisfied with how they were doing and felt that it was not necessary to make big changes in their diet. In addition, they talked about giving themselves some leeway when they wanted to eat something unhealthy like French fries or chocolate. They used terms like “normal”, “balanced” and “standard diet” to describe their pregnancy diet. Similarly, some women indicated that food and eating during pregnancy should not feel like being on a diet. After being asked whether healthy eating during pregnancy was difficult, one woman responded:

I do not think it differs that much from normal healthy foods. Except that you should avoid raw dairy and meat etc. So I do think, if you emphasise that, healthy food is this and this and this … that holds true for everybody, it is not just if you are pregnant, then you are not allowed to eat this or that. (P1)

The women also frequently mentioned that pregnancy is characterized by mental pressures or hormones that make it difficult to eat healthy, such as experiencing cravings or hunger at night. Most women who experienced these difficulties said they were unable to control themselves and that this is also acceptable given that they were pregnant. While this may seem contradictory to their strong sense of responsibility for the child, the women talked about these situations as being two sides of the same coin. For example, an episode of unhealthy snacking could be balanced by eating more fruits and vegetables the next day. More importantly, restricting oneself during pregnancy to solely healthy eating was considered disempowering. It was therefore considered important by the women to not worry or stress about having an appetite for unhealthy foods during pregnancy:

Some days I am not hungry, then I eat just normal small portions. And on other days I just cannot seem to eat enough, and I am not worried about it too much. I think that it is a normal thing if your pregnant, just as long as you do not have a hungry day every day. (P9)

Finally, some women recognized that there can be a conflict between the motivation to eat healthfully and other interests, such as the wish to behave like a normal person or to join others in common social practices. For example, when talking about the possibility to reduce snacking during her pregnancy, one woman replied:

I also have children. Like in the weekend. Then I am eating chips with the girls, and salty snacks. So yes, no, I am not going to change that. Otherwise I would be just sitting there while the children are enjoying their chips. No, I will not change that. (P12)

### Interviewer’s reflections

Two reflections are offered on the conducted interviews in relation to the opportunities for empowerment. First, by creating an open atmosphere at the start of the interviews, the participating women were talkative and seemed to use the opportunity to discuss issues that were important to them at the start of the interview, even before the interviewer could ask the first question. They discussed many issues and experiences, such as illnesses, fears, previous pregnancies. Looking back at the interviews, these introductory talks seemed important in establishing a relationship between the interviewer and the participant and it opened up the possibility to have a real dialogue about the sensitive topics of pregnancy, food, and eating.

Secondly, although it was not the intention of the interviewer to offer nutritional advice, women were frequently interested in discussing dietary requirements and practical strategies to eat healthier. The interviewer made strong efforts to be supportive, understanding, and respectful to the pregnant women by offering encouraging feedback and being sensitive and empathetic to their experiences. The interviewer felt that the mind map exercise played an important role in the interview process, as it allowed the women to present their views on food and eating during pregnancy. In a number of interviews, the brainstorm and the exercise with the nine food cards also led the women to express new intentions for healthy eating, as was already demonstrated under theme 1. Although it remains a question whether these intentions are also implemented in everyday life, it does indicate that an open discussion can make a first step in empowerment towards healthy eating.

## Discussion

This study aimed to answer two research questions: 1) “What are the perspectives of low SES pregnant women on food and eating during pregnancy?” and 2) “What are the opportunities for empowerment towards a healthy dietary intake amongst low SES pregnant women?” Five different perspectives on food and eating emerged during the brainstorm with low SES pregnant women: 1) health-promoting foods and products; 2) challenges in relation to eating during pregnancy; 3) risky products; 4) strategies for healthy eating, and; 5) motivational aspects. In addition, four opportunities for empowerment towards a healthy dietary intake were identified: 1) searching balanced and personalized information; 2) developing strategies to implement healthy diets; 3) navigating the social environment, and; 4) dealing with different motivations. In general, the participating women in general demonstrated a strong motivation for healthy eating and felt responsible for the health of the baby. Moreover, our results demonstrate that pregnant women do make dietary changes for the health of the baby, which is in line with other research reporting that women make dietary changes during pregnancy (Forbes et al., 2018).

**Figure 1.**
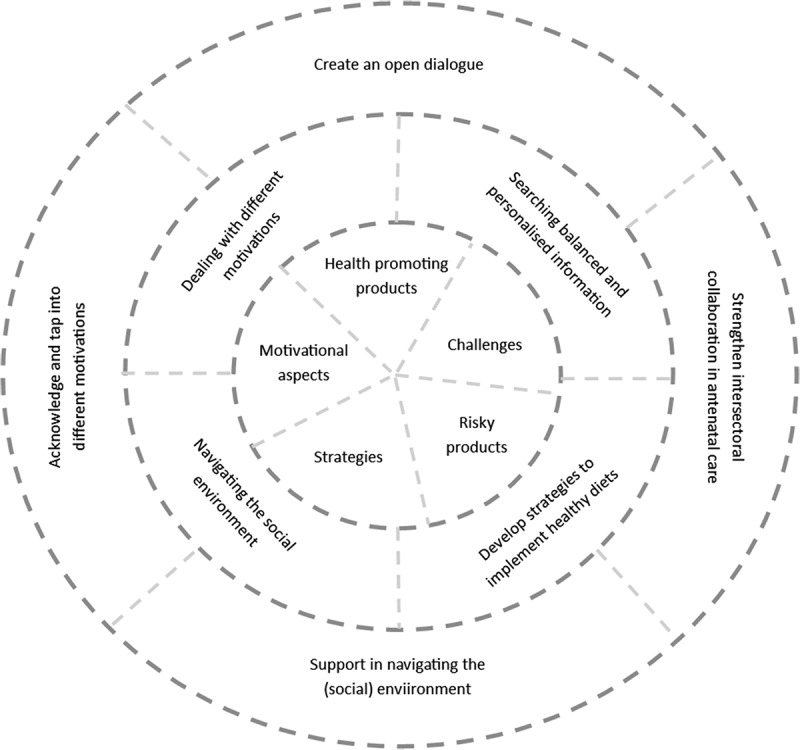
Overview of study results and recommendations

**Figure 2.**
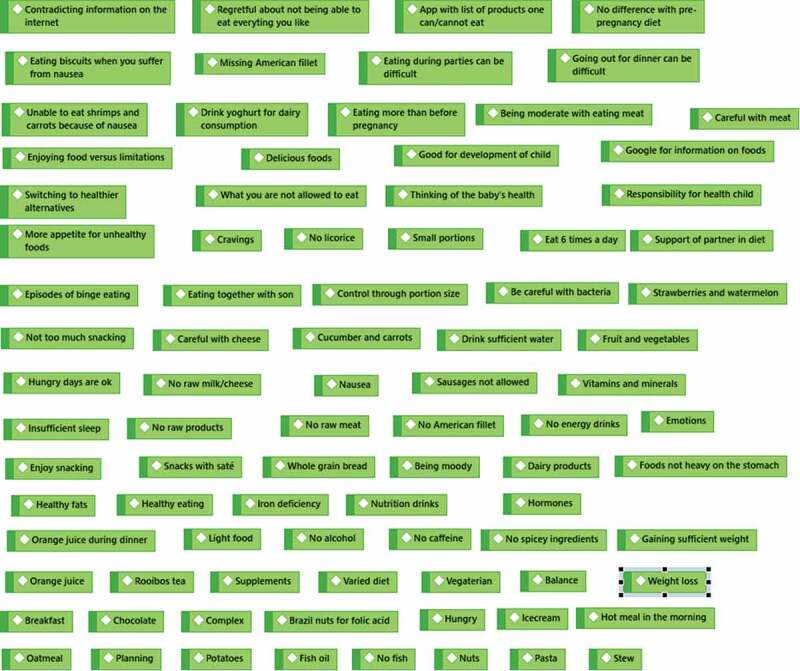
Step 1 of the qualitative content analysis: manifest content codes

The results from this study offer a number of suggestions for improving the support that health professionals in antenatal care can offer to low SES pregnant women. First, based on the results of research question 1, we suggest that the diverse perspectives of pregnant women on food and eating during pregnancy should be addressed in the dietary advice that is provided (i.e., the “what” of dietary advice). With respect to the results of research question 2, we offer four recommendations on “how” health professionals could support pregnant women in empowering themselves towards a healthy dietary intake. Figure 1 provides an overview of the results from both research questions and the recommendations derived from these results. The dotted lines in Figure 1 indicate that there is overlap and interaction between the various elements of the figure. In the remainder of the discussion we reflect on each of these recommendations in relation to the study results Figure 2 and 4.

**Figure 3.**
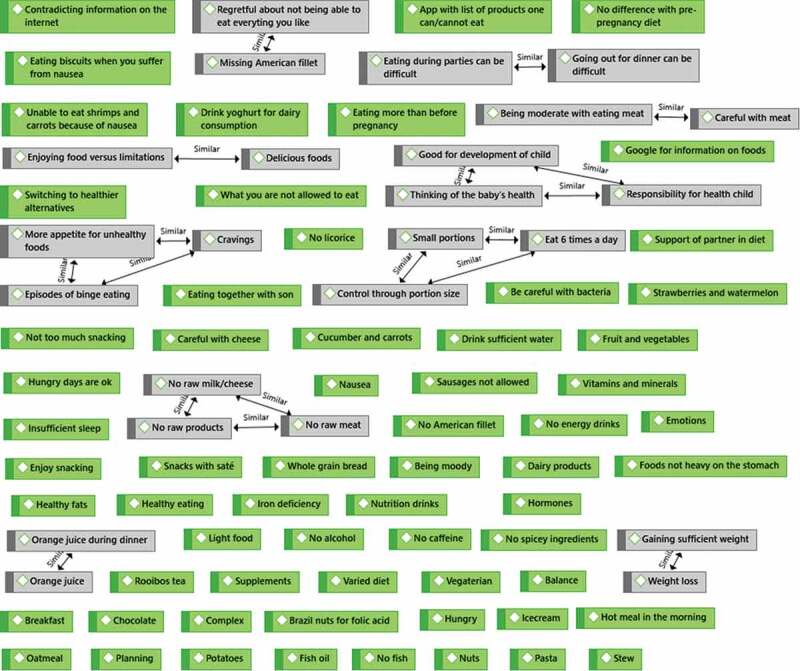
Step 2 of the qualitative content analysis: identifying overlapping codes

**Figure 4.**
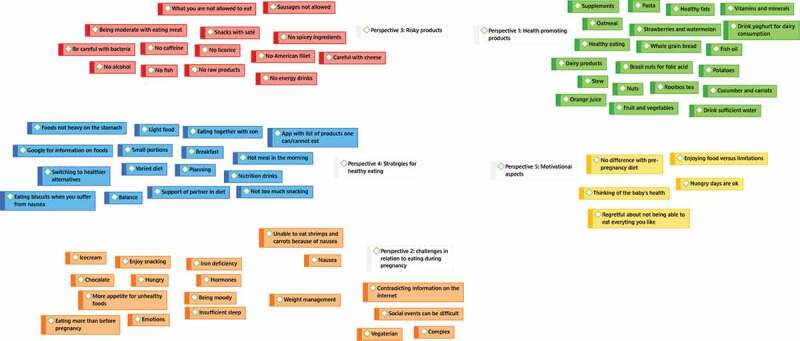
Step 3 of the qualitative content analysis: identifying common themes and grouping codes

The core of Figure 1 are the diverse perspectives of pregnant women on food and eating during pregnancy. Understanding pregnant women’s perspectives on problems and issues is an essential first step in the empowerment process, as it signals what is important to the women (Tengland, 2012). For example, in this study, we found that the two most predominant perspectives on food and eating during pregnancy related to health-promoting foods and products, and the challenges to eat healthy during pregnancy. Supporting low SES pregnant women requires health professionals, such as midwives, to address these perspectives in nutrition advice. Yet, studies have frequently demonstrated that midwives pay little attention to nutrition during antenatal care practices and when they do, they tend to focus on food risks (Garnweidner et al., 2013; Wennberg et al., 2013). The World Health Organization (2018) recommends providing positive nutritional counselling to all pregnant women and offers specific nutritional recommendations for a healthy dietary intake, including vitamin and mineral intake. A recent study amongst Australian antenatal care clinicians and pregnant women showed that knowledge about requirements for a healthy dietary intake was limited amongst pregnant women and that only 30% of the antenatal care clinicians had correct knowledge about these requirements (Lee et al., 2018). The limited attention for healthy eating was also acknowledged by the pregnant women in this study, who indicated that they received little information on health-promoting products and generally only after asking specific questions to the midwife. This signals that there is a great opportunity to increase the relevancy of the nutritional advice given to pregnant women by better matching their perspectives on food and eating during pregnancy. In order to provide adequate nutrition advice, it is also important that midwives are equipped to provide this advice and that nutritional guidelines are easily accessible and understandable for midwives, who generally do not consider themselves to be nutrition experts (Beulen et al., in preparation).

The second circle in Figure 1 reflects the opportunities for empowerment that were identified in the second research question. These formed the basis for four suggestions on “how” pregnant women can be supported in empowering themselves towards a healthy dietary intake, reflected in the third and outer circle of Figure 1. First, the results of this study signal the importance of engaging in an open dialogue with pregnant women to identify meaningful issues in relation to healthy eating. Scholars have pointed out that an open dialogue with room for diverse perspectives and interests is indeed needed to move towards empowerment (Frederick & Lee, 2019). An open dialogue, in line with Freire’s pedagogical ideas, creates a common understanding between patient and health professional and a basis for collaboration, acknowledgement and legitimacy (Tveiten & Knutsen, 2011). This dialogue should be part of a reciprocal and dynamic relationship in which patients and professionals create trust and work together towards a shared goal. This current study indicates that personalized information is strongly appreciated by pregnant women, as they are confronted with pregnancy-related challenges such as a dislike for specific products or nausea that require specific adaptations in their eating patterns. In addition, the women in the current study expressed the need for information on why certain choices are considered (un)healthy, to be able to draw their own conclusions and make autonomous decisions. This finding is in line with a large European study that demonstrated that pregnant women preferred personalized information about their risk of gestational diabetes and tailored advice regarding lifestyle modifications (Jelsma et al., 2016). However, creating an open dialogue as a health professional is not self-evident (Koelen & Lindström, 2005; Stans et al., 2018; Tveiten & Meyer, 2009), and it requires that health professionals themselves become empowered as well.

Secondly, the pregnant women preferred to receive support in the challenges they were facing regarding healthy eating. These diverse challenges included physical symptoms of pregnancy, dislike for specific healthy products during pregnancy, dealing with time pressure, and dealing with social situations. As midwives have little time to offer personalized support (McCann et al., 2018) and frequently indicate that they lack sufficient dietary knowledge to provide nutritional advice (Arrish et al., 2014), it might be useful to strengthen intersectoral collaboration of midwives with other health professionals, such as with dietitians or nutritionists. A study by Wilkinson and McIntyre (2012) showed that a one-hour dietitian-led behaviour change workshop led to a significant increase in fruit consumption and vegetable intake in comparison to a control group that received a booklet with evidence-based information on healthy eating during pregnancy. A personalized dietary intervention delivered by a dietitian also proved more effective in limiting weight gain during pregnancy as compared to a general intervention promoting healthy eating (Di Carlo et al., 2014). Most women in the current study were open to the idea of consulting a dietitian as part of regular antenatal care consultations, especially when they were experiencing eating-related challenges during pregnancy. However, it is not common practice in the Netherlands to consult with a dietitian during pregnancy and midwives often only refer to dietitians in case of medical necessity (e.g., excessive weight gain) (Super et al., 2019). Low SES pregnant women experience barriers to visiting a dietitian, including financial difficulties, stigma associated with going to a dietitian, and being unfamiliar with the possibility to consult a dietitian for pregnancy-related questions. As such, there is much to be gained from exploring how dietitians could empower low SES pregnant women towards a healthy dietary intake in close collaboration with other antenatal care professionals.

Thirdly, the social environment was both a facilitating as well as a hindering factor in healthy eating during pregnancy. As a facilitating factor, the social environment (i.e., one’s partner, children, friends,and relatives) could support pregnant women by offering nutritional information and practical tips on healthy eating during pregnancy, and by facilitating successful dietary changes. In their study with low-income pregnant women, Fowles et al. (2012) found that women with a diet quality below the median reported to have less social support than women with diet quality above the median. Receiving support for and approval of making healthy choices is key in making sustainable dietary changes. Conversely, the social environment was a hindering factor for several participants who mentioned that they felt that their eating and drinking behaviours during pregnancy were monitored by their relatives, friends, or even strangers. The women felt that they could be blamed for an unhealthy child if they had an unhealthy dietary intake during their pregnancy. At the same time, however, they felt that they were allowed some leeway because they were pregnant and should not be blamed if they occasionally snacked or had food cravings. An overemphasis on the mother’s responsibility for a healthy child through healthy eating can become disempowering when the internal conflict between “being a good mother” and “giving leeway” increases stress, anxiety, and feelings of incompetence. In this respect, it is important to recognize that attributing health behaviours solely to individual choice and decision-making fails to recognize the influence of the social and environmental determinants on health (Belon et al., 2016; Tengland, 2012; Williams et al., 2012). Indeed, the results of this study suggest that pregnant women need to be supported in navigating the pressures of their social environment. Perhaps more importantly, failing to recognize that there are also insurmountable barriers in their lives to implement a healthier diet can prove disempowering. *“People become empowered when they learn to distinguish between the current situation and their future possibilities, but always with the recognition of existing vulnerabilities and impossibilities* [quote translated from Dutch] (Boumans, 2012, p. 47).” Moreover, overemphasis on food risks and the urgency of a healthy diet during pregnancy may lead to stronger feelings of vulnerability and shame, and prove counter-productive. In order to support the empowerment of pregnant women towards a healthy dietary intake, health professionals, such as midwives or dietitians, should also map the social environment in which pregnant women live to identify opportunities and challenges for healthy eating.

Fourthly, the results of this study indicate that women faced conflicting motivations when it came to eating healthy despite holding a general strong motivation for healthy eating in general. A similar result has been found in a study amongst low-income parents where psychosocial related child feeding goals (e.g., maintaining family relationships) and nutrition-related child feeding goals (e.g., healthy diets) created tension (Schuster et al., 2019). The authors conclude that even though parents exhibit motivation to achieve healthy eating goals, the presence and persistence of psychosocial goals often lead to less-healthy practices. It is important for health professionals in antenatal care to acknowledge and recognize the presence of conflicting types of motivations to help pregnant women to develop strategies to deal with these different motivations (Schuster et al., 2019). Low SES patients may also have fatalistic beliefs and mindsets which can hinder the opportunity to create an open dialogue and to work towards empowerment (Frederick & Lee, 2019). Although the results of this study indicate that low SES pregnant women are generally motivated to have a healthy dietary intake, more research is needed to explore how this motivation is translated into behaviour and how health professionals can tap into this motivation to empower them towards a healthy dietary intake.

In summary, empowering low SES pregnant women towards a healthy dietary intake requires health professionals in antenatal care to reconsider their role. Health professionals can take various roles in the empowerment of individuals or communities, ranging from more traditional roles like “a rescuer” (e.g., providing necessary medical support) or “a provider” (e.g., providing medical advice), to more innovative roles like “a facilitator” (e.g., bringing people together) or “an advocate” (e.g., lobbying for changes in the environment) (Toomey, 2011). The more traditional roles tend to be disempowering as they result in vertical relations between health professional and patient and fail to recognize environmental influences on behaviour. Health professionals in antenatal care should thus shift towards more innovative roles in which there is an increased focus on changing the environment(s) in which people live. Further research is needed to explore how health professionals can be best supported to rethink their role in antenatal care and what is needed for them to engage in an empowerment process with pregnant women.

### Strengths and limitations

This study is the first to explore the perspectives of low SES pregnant women on food and eating during pregnancy and to identify opportunities for empowerment towards a healthy dietary intake. The co-design meetings were helpful in developing the interview questions and activities, and increased the quality and validity of data collection. The use of mind maps in the interviews was a strength of this study as it provided valuable insights on the participants’ experiences as well as created an open atmosphere that allowed for further exploration of opportunities for empowerment. Although it was not the aim of the interviews to engage in a process of empowerment with the participants, it nevertheless seemed as though there were steps taken towards empowerment. For example when discussing the recommended intake for pregnant women for specific product groups, some women indicated to have learned something new and expressed the intention to make a dietary change based on this knowledge.

Although data saturation was reached, the low number of participants is nevertheless a limitation of this study. The study makes a strong case for rethinking the way nutritional support is offered to low SES pregnant women. However, more research is needed on how to optimize this support within concurrent antenatal care practices. Given that perspectives on food and eating are coloured by culture and social norms (Bailey, 2017; König et al., 2017), it would be valuable to consider the differences in meanings attached to food and eating during pregnancy across countries or between ethnic groups, and to explore how these differences translate into empowerment strategies of health professionals to promote the healthy dietary intake during pregnancy.

## Conclusion

Our study indicates that women hold very diverse perspectives regarding food and eating during pregnancy, signalling the need to adjust dietary support by taking these perspectives into account. In addition, the findings of this research suggest that health professionals, such as midwives, have a major opportunity to expand their role in empowering low SES pregnant women to consume a healthy dietary intake. Several opportunities for empowerment have been identified: searching balanced and personalized information, developing strategies to implement healthy diets, navigating the social environment, and dealing with different motivations. Given that pregnant women are generally motivated to make dietary changes, reconsidering antenatal care practices to engage in the empowerment process with pregnant women may be an effective step, thereby overcoming important challenges that low SES pregnant women face during pregnancy.

## Supplementary Material

Supplemental Material
