# Supplementary figures and images for: Understanding empowerment for a healthy dietary intake during pregnancy
Source: Int J Qual Stud Health Well-being. 2020 Dec 14;16(1):1857550. doi: 10.1080/17482631.2020.1857550 (PMC7738301; doi:10.1080/17482631.2020.1857550)

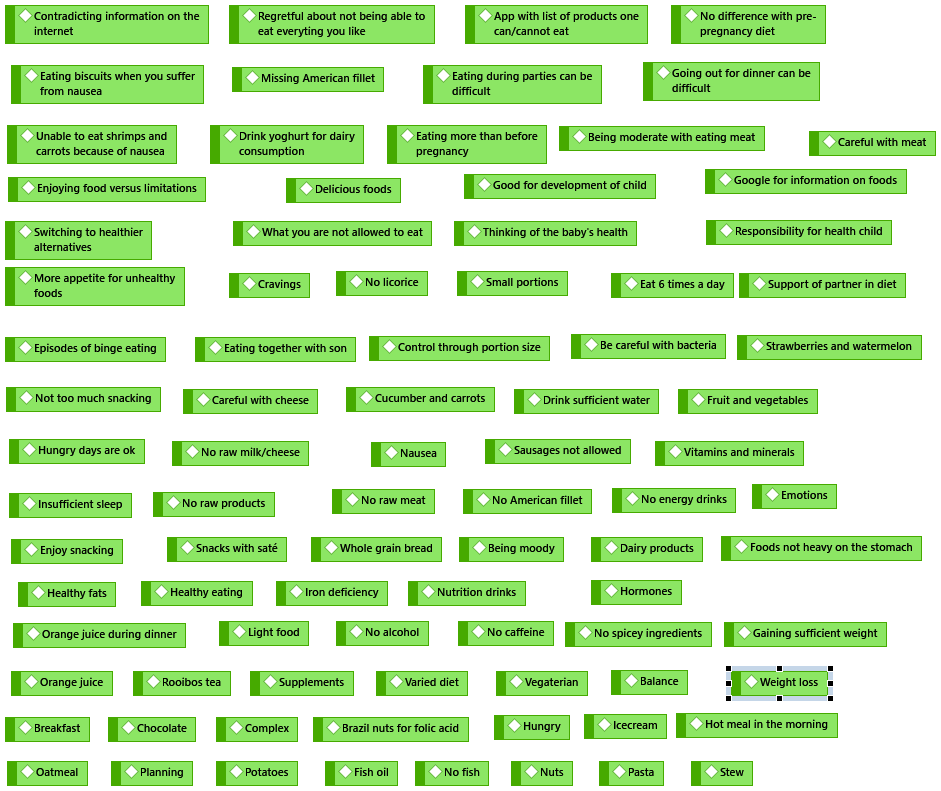

Supplement: Supplemental Material [file ZQHW_A_1857550_SM4518.zip › Supplementary/Figure 2 _Supplementary file _Step 1 manifest content.tif]

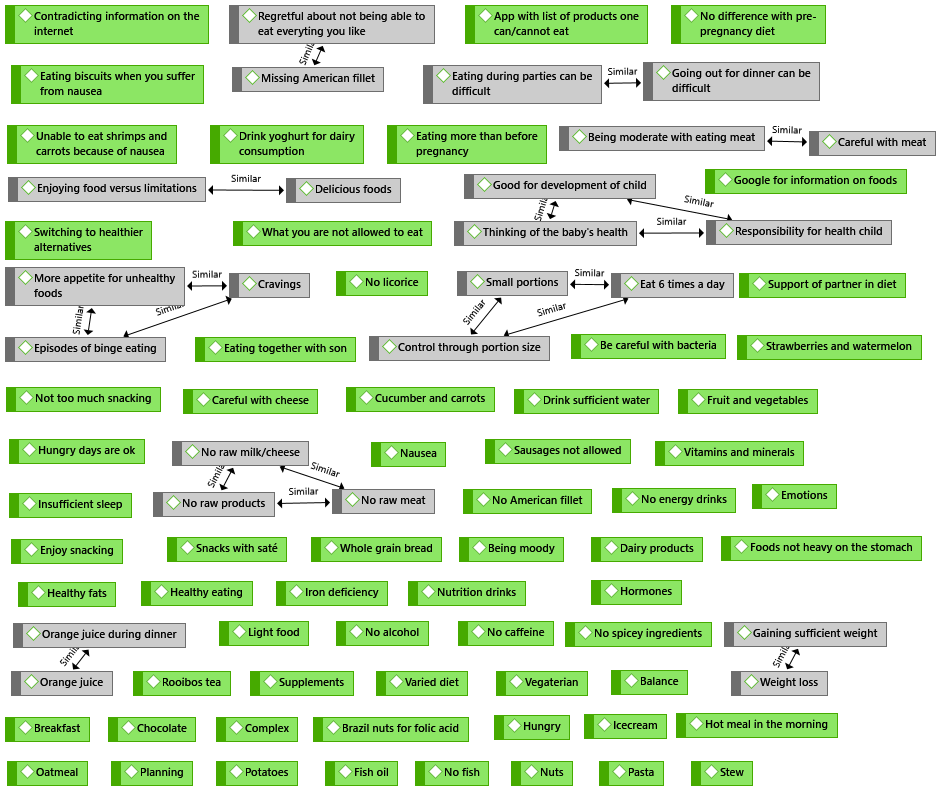

Supplement: Supplemental Material [file ZQHW_A_1857550_SM4518.zip › Supplementary/Figure 3 _Supplementary file _Step 2 grouping overlapping codes.tif]

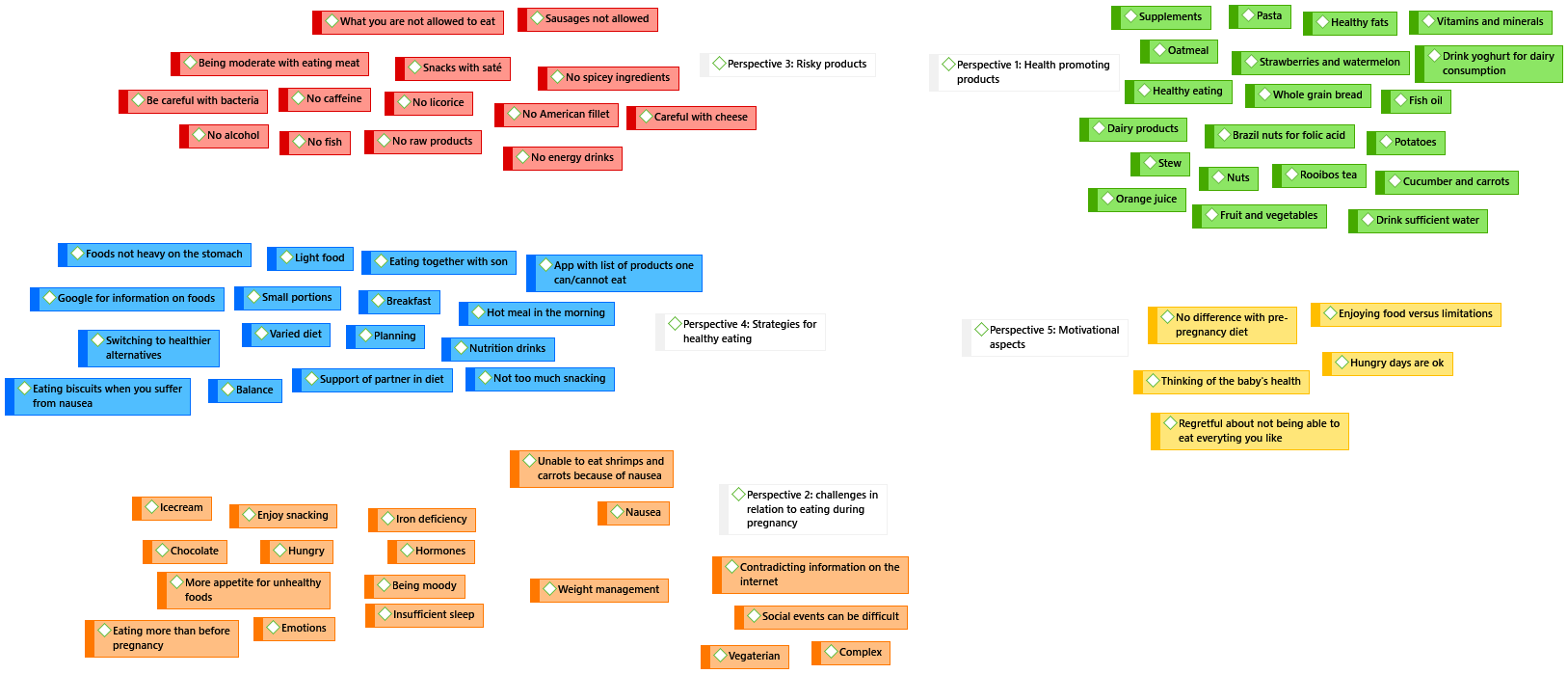

Supplement: Supplemental Material [file ZQHW_A_1857550_SM4518.zip › Supplementary/Figure 4_Supplementary file_Step 3 common themes.tif]
